# Participant’s treatment guesses and adverse events in back pain trials: Nocebo in action?

**DOI:** 10.1177/17407745241276124

**Published:** 2024-09-13

**Authors:** Javier Muñoz Laguna, Hyangsook Lee, Eduard Poltavskiy, Jeehyoung Kim, Heejung Bang

**Affiliations:** 1 EBPI-UWZH Musculoskeletal Epidemiology Research Group, University of Zurich and Balgrist University Hospital, Zurich, Switzerland; 2 Epidemiology, Biostatistics and Prevention Institute (EBPI), University of Zurich, Zurich, Switzerland; 3 University Spine Center Zurich (UWZH), Balgrist University Hospital and University of Zurich, Zurich, Switzerland; 4 Department of Science in Korean Medicine, College of Korean Medicine, Graduate School, Kyung Hee University, Seoul, Korea; 5 Independent Researcher, Sacramento, California, United States; 6 Department of Orthopedic Surgery, Seoul Sacred Heart General Hospital, Seoul, Korea; 7 Division of Biostatistics, Department of Public Health Sciences, University of California, Davis, California, United States

Randomized controlled trials (RCTs) of back pain often prioritize efficacy or effectiveness outcomes. To determine safety, back pain RCTs, monitor adverse events. Participants’ guesses about their assigned intervention are rarely reported, and quantitative assessments of blinding success are sparse. Blinding data are challenging to locate, retrieve, or pool. Freed et al.^1^ conducted a meta-analysis of 40 back pain RCTs, published between 2000 and 2019 which reported participants’ treatment guesses. A main finding was the association between correct guesses and the size of treatment effects. Even though blinding may not always be crucial to study objectives,^2,3^ patient-reported outcomes are susceptible to bias introduced by participants’ correct beliefs or perceptions about intervention assignment.^4,5^

Here, we explored associations between participants’ treatment guesses and adverse events in back pain RCTs updating the search of Freed et al.^1^ We hypothesize that “active” intervention guesses are associated with higher adverse event rates, whereas “control” intervention guesses are associated with lower rates—a commonly held, although untested belief. We also hypothesize that satisfactory blinding is associated with fewer adverse events.

Freed et al.^1^ analyzed 40 back pain RCTs published between 2000 and 2019. Two eligible studies not included in that review were identified after that publication. From 2020 to 2023, we searched “back pain” and “blind” in PubMed for screening. Similar searches were conducted in Ovid and Embase. Eligible articles were subjected to word searches on blinding and adverse events.

Participants’ intervention assignments (“Active” vs “Control”) were tabulated against treatment guess data (“Active” vs “Control” vs “Don’t know”). Then, a blinding index (BI) was computed.^1,5–7^ Credibility/expectancy scores were reported in some RCTs and converted to a BI via linear transformation (see Supplemental materials). In addition, we extracted treatment modality and timing of blinding assessment.^8^ For safety outcomes, we extracted the most prominently reported adverse event for primary analysis and another one for secondary/sensitivity analyses, whenever available. Adverse events were analyzed in terms of count and rate (i.e., count/total sample size).

We calculated Pearson correlation coefficients and fitted locally estimated scatterplot smoothing (LOESS) with confidence limits between arm-specific BI and adverse events. Next, we repeated these analyses for the correlation between “sum BI” and “between-arm differences in adverse events.” BI can be interpreted as the proportion of correct guesses *beyond chance* within an arm with 0 indicating a “random guess”, equivalently, 50:50 correct: incorrect; 1 indicating complete correct guess; and −1 indicating complete incorrect guess.^1,5,6^ Sum BI represents the degree of discrepant guesses between arms, with 0 indicating no discrepancy, for example, all participants in both arms guessing they received the active treatment. Satisfactory blinding was operationalized as two different scenarios: (1) (|BI_active_|≤ c and |BI_control_|≤ c; “random guess”), or (2) (|BI_sum_|≤ c; “wishful thinking”).^1,5,6^ Two cutoffs c ={0.2,0.3} were chosen.^6,9^

PubMed identified 230 publications for screening, supplemented by other search approaches. Twelve RCTs meeting eligibility criteria were identified for the period between 2020 and 2023 (Supplemental materials). Among 54 back pain RCTs-with participants’ treatment guess data, 45 reported adverse events.

Correlation coefficients between BI and adverse events in active arms were −0.17 and 0.01 using count and rate, respectively. The corresponding correlations for control arms were 0.28 and 0.15. For sum BI and between-group adverse event difference, the corresponding values were 0.31 (95% confidence interval (CI): 0.02–0.55) and 0.31 (0.01–0.55); see Figure 1.

**Figure 1.**
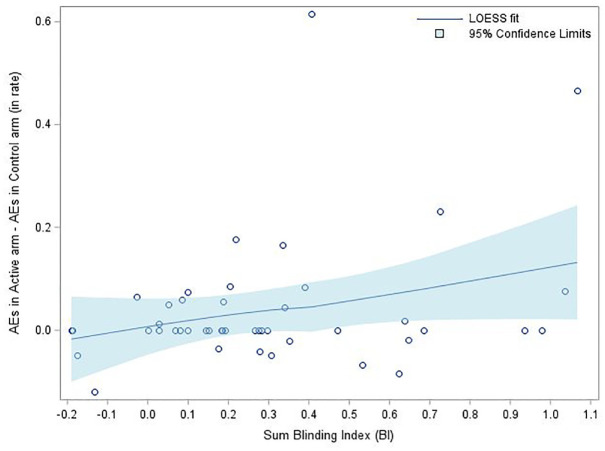
Discrepant guesses between arms (sum BI) and adverse event rates. Sum BI and between-arm adverse event rate differences (adverse event rates [active] - adverse event rates [control]) are displayed. Y-axis represents adverse event rate differences; the total number of adverse event cases were divided by total sample size within an arm, then the value of between-arm difference was calculated. Magnitude of discrepant guesses between arms (in X-axis) was measured by summed BIs (sum BI); BI_sum_ = BI_active_ + BI_control_, where sum BI closer to 0 indicates that participants in both arms believe they received the same treatment. For example, BI_sum_ = 0 can be “wishful thinking” when both groups believe they received the active treatment, or “pessimistic thinking” when both groups believe they received control; both scenarios result in BI_sum_ = 0, although “wishful thinking” is more common in practice.^1,5,6^ The regression line is fitted by LOESS with 95% confidence limits, based on the method of Cleveland and Grosse (1991). A total of 45 trials with BI and adverse event data are included. Pearson correlation coefficient is 0.31 (95% CI: 0.02–0.55). Correlation is 0.31 when count is used instead of rate, and 0.29 when weighted by total sample size for each trial. SAS version 9.4 was used for analysis and graphics, including the LOESS procedure (SAS Institute, Cary, NC).

Using 0.2 and 0.3 cutoffs, 22 and 31 out of 45 RCTs were classified as reaching “satisfactory blinding,” respectively. In the within-arm exploration, adverse events were not systematically different between the RCTs classified as “satisfactory blinding” and their not-satisfactory counterpart (i.e., the complement set). In contrast, the between-arm adverse event difference was low in the satisfactory blinding RCT group. With the 0.2 cutoff, the mean event counts were 0.5 for the satisfactory group versus 2.7 for the not-satisfactory counterpart, while the respective rates were 0.0 versus 0.08. Results tended to be robust to alternative adverse events and cutoffs; see Supplemental materials.

We explored associations between participants’ treatment guesses and adverse events in back pain RCTs. Our first hypothesis was not supported—active and control treatment guesses were not significantly correlated with increased and decreased adverse events, respectively. Correlations between arm-specific BIs and adverse events were weak, but somewhat in opposite directions, compared to correlations between arm-specific BIs and size of treatment effects reported previously.^1^ When using sum BI as a measure of study-level blinding, lower between-arm difference in adverse events was observed among satisfactorily blinded RCTs.

Our interpretations warrant caution due to marked clinical heterogeneity among included RCTs. Participant characteristics, types and timing of treatment guess and safety outcomes also varied. Other RCTs—outside back pain—have found associations between patient blinding and adverse events.^10^ Our findings provide a quantitative assessment of blinding and adverse events in back pain trials, suggesting that some adverse events could be due to participants’ treatment guesses about their assigned intervention. Hence, adverse events in sham- or placebo-control arms could be predominantly contextual or non-specific—namely, *nocebo* effect (i.e., *placebo* side effect).

Limitations of our study should be noted. First, stratified, subgroup, or adjusted analyses were not feasible; thus, sources of heterogeneity were not elucidated. Second, our data searches and review processes could not be as systematic as in traditional meta-analyses, owing to rarely collected and hard-to-reach blinding-related data, often buried within text or file drawer. Third, our operationalization of “satisfactory blinding” is numerical and ad hoc, so misclassification is plausible, when *true* status is hardly known.^6^ Last, we could not address temporality between blinding and adverse events.^11^

To our knowledge, this is the first exploration of participant’s treatment guesses and adverse events in back pain trials. This is noteworthy as reviews and meta-analyses on blinding generally rely on authors’ self-labeling of “single” or “double-blind”—especially, in title—rather than quantitative blinding assessments.^2,10^

Participant blinding and between-group comparisons can be important for safety outcomes, beyond efficacy or effectiveness outcomes. In the current patient-centered era with an emphasis on patient-reported outcomes, our findings provide empirical evidence that more discrepant treatment guesses could be associated with larger between-group differences in adverse events. Back pain trialists should foster blinding assessments and implementation procedures—including credible sham- or placebo-controls—and mitigate sources of bias in the evaluation of safety outcomes.^12^

## Supplemental Material

sj-docx-1-ctj-10.1177_17407745241276124 – Supplemental material for Participant’s treatment guesses and adverse events in back pain trials: Nocebo in action? Supplemental material, sj-docx-1-ctj-10.1177_17407745241276124 for Participant’s treatment guesses and adverse events in back pain trials: Nocebo in action? by Javier Muñoz Laguna, Hyangsook Lee, Eduard Poltavskiy, Jeehyoung Kim and Heejung Bang in Clinical Trials
