# Supplementary material for: Participant’s treatment guesses and adverse events in back pain trials: Nocebo in action?
Source: Clin Trials. 2024 Sep 13;21(6):759–62. doi: 10.1177/17407745241276124 (PMC11528854; doi:10.1177/17407745241276124)
Supplement: sj-docx-1-ctj-10.1177_17407745241276124 – Supplemental material for Participant’s treatment guesses and adverse events in back pain trials: Nocebo in action? [file sj-docx-1-ctj-10.1177_17407745241276124.docx]

**Appendix 1.** Search processes and data transformation

1. Search processes and results

In August 2023, we searched “back pain”[MeSH Terms] AND “blind” AND (“2020/01/01”[Pdat]: “2023/12/31”[Pdat]) in PubMed as a *primary* search source. One author (HB) skimmed each publication (or abstracts if full text was not publicly available), along with word searches for blinding or treatment guess data (e.g., “blind”, “mask”, “guess”, “believe/belief”, “perceive/perception”, “blinding assess”, “blinding index”, “credibility”, “expectancy”, etc.) and for adverse events (AEs) or serious adverse events (SAEs) (e.g., “adverse” “side”, “safe”, and “complic”). Of note, our search syntax yields the same number of publication/entries as “back pain”[MeSH Terms] AND (“Single-Blind Method”[MeSH Terms] OR “Double-Blind Method”[MeSH Terms] OR “blind”) AND (“2020/01/01”[Pdat]: “2023/12/31”[Pdat]) does. Also, masked methods as well as single and double blinded studies are already included under blind methods with “blind” through MeSH terms and automatic term mapping mechanisms in PubMed.

Next, we used Ovid-Medline and Embase-Medline as secondary search engines, where we only intended to identify studies which were not already among the studies found by PubMed. With Ovid, search was done with “(back pain).mp” and Single-Blind Methods/ or Double-Blind Methods/ and year=”2020-2023”. With Embase, we used (‘back pain’/exp OR ‘back pain’) AND (‘blind’/exp OR blind) AND [2020-2023]/py AND (‘single blind procedure’/de OR ‘double blind procedure’/de) AND ‘randomized controlled trial’/de AND ‘low back pain’/dm AND (‘article’/it OR ‘article in press’/it). HB repeated the same skimming for the potential candidate trials identified. Additionally, <https://classic.clinicaltrials.gov/ct2/show/results> was searched for adverse event data, whenever these data were not clearly reported in publications.

Then another author (JML) independently conducted a random search and data check. HB and JML also contacted authors for required data (e.g., some authors did not report treatment assignment guess data or adverse events for each arm or timing of blinding assessment clearly; in some instances, the authors published the trial protocol but a final report was not available). They also contacted other trialists who published in the back pain field and performed blinding assessments in the past in the spirit of identifying recent eligible trials reporting blinding data. They did data checks and extractions, and variable classification and tried to reach consensus. When there was uncertainty, the two authors discussed with the third author (JK) or contacted the original authors of the publications. References in related systematic reviews published were also checked. All authors reviewed the final dataset. On December 31, 2023, PubMed, Ovid and Embase were searched again to check for any new study.

To summarize, as of December 31, 2023, 160 papers were identified by PubMed (primary source); 207 by Ovid and 212 by Embase for candidate screening. Then 7 qualified studies were found from PubMed; 1 additional from Ovid; and 1 additional from Embase. Additional 2 were found by contacting trialists in back pain and/or from systematic reviews. As a result, out of 53 (=40+2+11, where 2 belonged to 2000-2019) back pain trials with treatment guess data for years 2000-2023, a total of 44 trials also reported adverse event data.

After we received reviews from the journal, we repeated the search again once more on May 31 2024. PubMed, Ovid and Embase found 230, 209 and 360 articles, respectively. Through these updated searches, we could include 1 additional trial qualified, so a total of 45 trials in our final analysis.

1. Data transformation

We converted credibility/expectation scores reported in 4 trials (Nguyen et al., Sung et al., Dougherty et al., Bagg et al.) to BI using a linear transformation. This pragmatic transformation considered the similarity of the two constructs, as well as potential compromises to psychometric properties:

For Nguyen et al.’s and Sung et al.’s studies, BI_active_ = [2*(X-1)/8]-1;

for Dougherty et al, BI_active_ = [2*(X-1)/9]-1; and

for Bagg et al., BI_active_ = [2*(X)/48]-1,

where X is the reported mean value. For control arm, we multiplied the transformed BI by -1.

**Appendix 2.** Study overview of included trials

Trials conducted between 2000 and 2019: A total of 40 trials included in Freed et al. (2021) are summarized in their Supplements (table and references). Of note, after the publication of Freed et al., our authors found 2 eligible trials that had been omitted; Bialosky et al. (2014) and Dougherty et al. (2014), and that 33 studies among these 42 also collected or reported adverse event information in publication or registration website.

Trials conducted between 2020 and 2023: New searches found a total of 12 eligible trials with blinding and adverse event information. See Table S1 and references below.

References of 2+12 newly included studies (in alphabetical order):

- Bagg et al. Effect of graded sensorimotor retraining on pain intensity in patients with chronic low back pain. A randomized clinical trial. JAMA 2022; 328(5): 430-439.
- Bialosky et al. Spinal manipulative therapy–specific changes in pain sensitivity in individuals with low back pain (NCT01168999). J Pain 2014; 15(2): 136-148. And <https://classic.clinicaltrials.gov/ct2/show/results/NCT01168999?view=results>
- Dougherty et al. Spinal manipulative therapy for chronic lower back pain in older veterans: A prospective, randomized, placebo-controlled trial. Geriatric Orthop Surg Rehabil 2014; 5(4): 154-164.
- Ehsani et al. Transcranial direct current stimulation over dorsolateral prefrontal cortex improves postural stability in non-specific chronic low back pain patients with high fear of pain: A randomized sham-controlled trial. Eur J Neurosci 2023; 58: 3315-3329.
- Garcia et al. Durability of the treatment effects of an 8-week self-administered home-based virtual reality program for chronic low back pain: 6-month follow-up study of a randomized clinical trial. J Med Internet Res 2022; 24(5): e37480.
- Gilligan et al. An implantable restorative-neurostimulator for refractory mechanical chronic low back pain: A randomized sham-controlled clinical trial. Pain 2021; 162: 2486-2498. And <https://classic.clinicaltrials.gov/ct2/show/results/NCT02577354?view=results>
- Gonzales et al. Validation of a sham novel neural mobilization technique in patients with non-specific low back pain: A randomized, placebo-controlled trial. Musculoskelet Sci and Pract 2021; 53: 102378.
- Kong et al. Effect of electroacupuncture vs sham treatment on change in pain severity among adults with chronic low back pain. A randomized clinical trial. JAMA Netw Open 2020; 3(10): e2022787.
- Koppenhaver et al. Effect of dry needling on lumbar muscle stiffness in patients with low back pain: a double blind, randomized controlled trial using shear wave elastography. J Man Manip Ther 2022; 30(3): 154-164.
- McPhee and Graven-Nielsen. Medial prefrontal high-definition transcranial direct current stimulation to improve pain modulation in chronic low back pain: A pilot randomized double-blinded placebo-controlled crossover trial. J Pain 2021; 22(8): 952-967
- Muñoz Laguna et al. Blinding assessment of manual therapy interventions of the back in Swiss graduate students: a blinding feasibility randomized controlled trial. 2023. <https://www.researchgate.net/publication/370176766>.

[Note: this paper was published in a peer-reviewed journal in 2024 after submission of this manuscript

<https://chiromt.biomedcentral.com/articles/10.1186/s12998-023-00524-x>]

- Nguyen et al. Effect of osteopathic manipulative treatment vs sham treatment on activity limitations in patients with nonspecific subacute and chronic low back pain. A randomized clinical trial. JAMA Intern Med 2021; 181(5): 620-630.
- Sung et al. Efficacy and safety of thread embedding acupuncture combined with acupuncture for chronic low back pain. A randomized, controlled, assessor-blinded, multicenter clinical trial. Medicine 2020; 99(49): e22526.
- Yue et al. Effectiveness and safety of intrathecal morphine for percutaneous endoscopic lumbar discectomy under low-dose ropivacaine: A prospective, randomized, double-blind clinical trial. Spine J 2023; 23: 954-961.

**Table S1.** List of included trials

| **Study & Year** | **N** | **Treatment  (Active, Control)** | **Timing of**  **blinding assessed** | **AE or SAE (Primary/Secondary)** |
| --- | --- | --- | --- | --- |
| Kerr 2003 | 30 | Verum acupuncture | Later | Side effect |
|  | 30 | Placebo transcutaneous electrical nerve stimulation |  |  |
| Pauza 2004 | 37 | Intradiscal electrothermal therapy | Early | AE |
|  | 27 | Sham therapy |  |  |
| Sator-Katzenschlager 2004 | 31 | Auricular electroacupuncture | Later | AE |
|  | 30 | Conventional manual  auricular acupuncture |  |  |
| Arden 2005 | 120 | Epidural corticosteroid injection | Later | Non-specific headache/Postdural puncture headache or nausea |
|  | 108 | Placebo injection |  |  |
| Hawk 2005 | 54 | Flexion-distraction chiropractic manipulation | Later | AE |
|  | 57 | Manual placebo |  |  |
| Brinkhaus 2006 | 147 | Verum acupuncture | Later | SAE/AE |
|  | 75 | Minimal acupuncture |  |  |
| Itoh 2006 | 13 | Trigger point acupuncture | Later | AE |
|  | 13 | Sham acupuncture |  |  |
| Atkinson 2007 | 52 | Desipramine | Later | Side effects that interfered daily function/Decreased salivation |
|  | 26 | Placebo (Benztropine) |  |  |
| Haake 2007 | 387 | Verum acupuncture | Later | SAE |
|  | 387 | Sham acupuncture |  |  |
| Cohen 2008 | 14 | Lateral branch denervation | Early | Serious complication |
|  | 14 | Placebo |  |  |
| Costa 2009 | 77 | Motor control exercise | Later | Mild adverse event |
|  | 77 | Placebo intervention |  |  |
| Kallmes 2009 | 68 | Vertebroplasty | Later | AE |
|  | 63 | Simulated procedure without cement |  |  |
| Kvarstein 2009 | 10 | Radiofrequency thermal disc therapy | Later | SAE |
|  | 10 | Sham treatment |  |  |
| Sigtermans 2009 | 30 | Ketamine | Early | Nausea/Vomit |
|  | 30 | Placebo |  |  |
| Munts 2010 | 10 | Intrathecal methylprednisolone bolus | Later | SAE/AE |
|  | 11 | Placebo |  |  |
| Wilkens 2010 | 125 | Glucosamine | Later | AE of any type/Resulting in study agent termination |
|  | 125 | Placebo |  |  |
| Pach 2011 | 51 | Homeopathic injection | Later | AE/Hematoma at injection site |
|  | 48 | Placebo injection |  |  |
| Cohen 2012 | 26 | Epidural etanercept | Early | Minor AE/SAE |
|  | 30 | Epidural saline |  |  |
| Patel 2012 | 34 | Lateral branch neurotomy | Early | Serious complication |
|  | 17 | Sham intervention |  |  |
| Cho 2013 | 65 | Verum acupuncture | Early | Total AEs/Temporarily worsened low back pain |
|  | 65 | Sham acupuncture |  |  |
| Walker 2013 | 92 | Chiropractic therapy | Later | Increased pain/SAE |
|  | 91 | Sham therapy |  |  |
| Biaolsky 2014 | 28 | Spinal manipulative therapy | Early | SAE/AE |
|  | 54 | Placebo (Standard or Enhanced) |  |  |
| Dougherty 2014 | 69 | Spinal manipulative therapy | Early | SAE |
|  | 67 | Sham |  |  |
| Fuentes 2014 | 30 | Interferential current therapy | Later | Increased pain/Other AE |
|  | 29 | Sham |  |  |
| Cohen 2015 | 73 | Epidural steroid injection | Early and Later | Total AE related to injection/Total AE related to drug treatment |
|  | 72 | Sham injection & Gabapentin |  |  |
| Luedtke 2015 | 67 | Anodal stimulation | Early | Minimal transitory side effect |
|  | 68 | Sham stimulation |  |  |
| Atkinson 2016 | 55 | Gabapentin | Later | Marked AE/Treatment emergent AE |
|  | 53 | Placebo |  |  |
| Clark 2016 | 61 | Vertebroplasty | Later | SAE |
|  | 59 | Placebo intervention |  |  |
| Braten 2019 | 85 | Amoxicillin | Later | SAE/AE |
|  | 91 | Placebo |  |  |
| Brutcher 2019 | 200 | Topical pain cream | Later | Side effect/Redness at 1 month |
|  | 195 | Placebo cream |  |  |
| Cohen 2019 | 64 | Fluoroscopy guided injection | Early | AE |
|  | 61 | Landmark guided injection |  |  |
| Gewandter 2019 | 16 | Gabapentin | Later | SAE/Diarrhea |
|  | 16 | Placebo |  |  |
| Kallewaard 2019 | 40 | Intradiscal methylene blue injection | Early and Later | SAE |
|  | 41 | Placebo injection |  |  |
| Kong 2020 | 59 | Electroacupuncture | Later | Reaction/Flairs |
|  | 62 | Sham treatment |  |  |
| Sung 2020 | 19 | Thread embedding acupuncture | Early | AE |
|  | 19 | Acupuncture |  |  |
| Gilligan 2021 | 102 | Therapeutic stimulation | Later | SAE/AE |
|  | 102 | Sham stimulation |  |  |
| Gonzales 2021 | 28 | Neural mobilization | Later | Side effect |
|  | 26 | Sham mobilization |  |  |
| McPhee 2021 | 12 | Active medial prefrontal cortex high-definition transcranial direct current stimulation (HD-tDCS) | Early or Later | Skin discomfort or hypersensitivity/ Headache |
|  | 12 | Sham HD-tDCS |  |  |
| Nguyen 2021 | 197 | Standard osteopathic manipulative treatment (OMT) | Later | AE/SAE |
|  | 197 | Sham OMT |  |  |
| Bagg 2022 | 138 | Graded sensorimotor retraining intervention | Later | SAE/AE |
|  | 138 | Sham procedure |  |  |
| Garcia 2022 | 94 | Virtual reality (EaseVRx) | Later | AE |
|  | 94 | Sham virtual reality |  |  |
| Koppenhaver 2022 | 30 | Dry needling | Later | Pain/SAE |
|  | 30 | Sham dry needling |  |  |
| Ehsani 2023 | 50 | tDCS | Early | Burning sensation or Pain |
|  | 25 | Sham stimulation |  |  |
| Muñoz Laguna 2023 | 11 | Active soft tissue mobilization | Early | AE |
|  | 13 | Control manual intervention |  |  |
| Yue 2023 | 45 | Intrathecal morphine | Later | Pruritus/Nausea or Vomit |
|  | 45 | Saline |  |  |

Listing is sorted by year first then alphabetically by first author’s last name.

Name of Treatment and Types of AE/SAE were reported as done in original publications.

AE: adverse events; SAE: serious adverse events.

N: number of participants (sample size) randomized; it can be substantially different from the sample size at the end of study.

*Early: immediately after randomization or the end of the first (or sole) treatment session. However, this distinction is not always clear so original authors were contacted and group discussion was used as needed.

**Table S2.** Distribution of adverse events for group by satisfactory blinding status

| Blinding Status* | Satisfactory Blinding  Group  Mean, Median (IQR) | Not Satisfactory Blinding Group  Mean, Median (IQR) |
| --- | --- | --- |
| Cutoff | 0.2 | |
| Number of trials | 22 | 23 |
| AE in Active arm – count  rate | 6.2, 3.5 (0, 6)  0.10, 0.05 (0, 0.2) | 8.0, 2 (1, 13)  0.15, 0.07 (0.01, 0.3) |
| AE in Control arm – count  rate | 5.7, 1.5 (0, 7)  0.10, 0.02 (0, 0.1) | 5.3, 2 (0, 5)  0.08, 0.04 (0, 0.1) |
| AE difference – count  rate | 0.5, 0 (0, 1)  0.00, 0 (0, 0.01) | 2.7, 0 (0, 7)  0.08, 0 (0, 0.09) |
| Cutoff | 0.3 | |
| Number of trials | 31 | 14 |
| AE in Active arm – count  rate | 6.7, 3 (0, 9)  0.09, 0.04 (0, 0.2) | 8.1, 2.5 (1, 19)  0.18, 0.07 (0.02, 0.4) |
| AE in Control arm – count  rate | 6.1, 1 (0, 7)  0.09, 0.03 (0, 0.1) | 4.3, 2 (0, 5)  0.08, 0.07 (0, 0.1) |
| AE difference – count  rate | 0.6, 0 (0, 1)  0.01, 0 (0, 0.01) | 3.9, 1 (0, 7)  0.11, 0.03 (0, 0.17) |

IQR: interquartile range, AE: adverse events.

Cutoff is for sum BI.

*Dichotomization of Satisfactory or not is numerical but ad-hoc.

**Table S3.** Sensitivity analysis: Table S2 for alternative^¶^ adverse events

| Blinding Status* | Satisfactory Blinding  Group  Mean, Median (IQR) | Not Satisfactory Blinding Group  Mean, Median (IQR) |
| --- | --- | --- |
| Cutoff | 0.2 | |
| Number of trials | 22 | 23 |
| AE in Active arm – count  rate | 7.4, 2.5 (1, 7)  0.17, 0.04 (0.01, 0.07) | 12.3, 2 (0, 14)  0.16, 0.08 (0, 0.3) |
| AE in Control arm – count  rate | 6.9, 2 (0, 7)  0.17, 0.03 (0, 0.1) | 11.0, 2 (0, 6)  0.13, 0.09 (0, 0.2) |
| AE difference – count  rate | 0.5, 0 (-1, 1)  0.00, 0 (-0.01, 0.01) | 1.7, 0 (-1, 3)  0.04, 0 (-0.01, 0.08) |
| Cutoff | 0.3 | |
| Number of trials | 31 | 14 |
| AE in Active arm – count  rate | 9.6, 2 (1, 8)  0.16, 0.05 (0.01, 0.1) | 10.4, 2 (0, 10)  0.18, 0.07 (0, 0.3) |
| AE in Control arm – count  rate | 8.9, 2 (0, 7)  0.16, 0.03 (0, 0.2) | 8.6, 2 (0, 4)  0.13, 0.08 (0, 0.1) |
| AE difference – count  rate | 0.7, 0 (-1, 2)  0.01, 0 (-0.01, 0.03) | 1.9, 0 (-1, 2)  0.06, 0 (-0.01, 0.08) |

IQR: interquartile range, AE: adverse events.

Cutoff is for sum BI.

¶For the primary analysis, we used AE or SAE reported in the most prominent place.

For this sensitivity analysis, we replaced primary AE by alternative AE (e.g., AE reported next

prominently) whenever reported.

*Dichotomization of Satisfactory or not is numerical but ad-hoc.
